# The Alzheimer's Association TrialMatch—Increasing awareness of all dementia trials

**DOI:** 10.1002/alz.71250

**Published:** 2026-03-09

**Authors:** Stephen Hall, Leticia Garcia, Marie Stahmer, Percy Griffin, Maria C. Carrillo, Rebecca M. Edelmayer

**Affiliations:** ^1^ Alzheimer's Association Chicago Illinois USA; ^2^ Former employee of the Alzheimer's Association Chicago Illinois USA

**Keywords:** Alzheimer's disease, awareness, clinical trials, dementia, recruitment

## Abstract

**BACKGROUND:**

Roughly 7.2 million older Americans are living with Alzheimer's disease (AD). Research participation remains one of the largest barriers facing dementia research advancements.

**METHODS:**

TrialMatch is a dementia research awareness tool. TrialMatch consolidates research opportunities to serve as an open‐access, centralized, and user‐friendly resource to identify research opportunities. Between August 2020 and June 2024, we tracked the number of research opportunities available, enrollment targets, and TrialMatch engagement.

**RESULTS:**

TrialMatch maintained a database of over 700 opportunities and received 18,802 calls and 122,461 web sessions, and provided 17,725 referrals to opportunities. An estimated 1,867,403 participants are needed to populate all the ongoing studies in the United States.

**DISCUSSION:**

By using a broad inclusion criteria, TrialMatch highlights the burden that AD/ADRD research faces with recruitment. While TrialMatch has demonstrated its ability to serve as an awareness tool, future analyses are needed to better evaluate the impact of the tool on enrollment.

## Highlights

- Over a 4‐year review period, the Alzheimer's Association's TrialMatch platform received 18,802 calls to its support line, 122,461 active web sessions, and provided 17,725 referrals to dementia research studies.
- Based on a review of all dementia research studies included in TrialMatch, a total of 1,867,403 participants are needed to complete all ongoing studies.
- The TrialMatch database contains enrollment data for 587 dementia research opportunities, which is far more than more restrictive analyses looking at clinical trials have analyzed previously.

## 1 INTRODUCTION

Despite recent advances in treatment, diagnosis, and risk reduction, Alzheimer's disease and Alzheimer's disease‐related dementias (AD/ADRD) remain a significant public health burden. Given the long duration of disease, people living with AD/ADRD may spend a significant amount of time in a state of severe disability and dependence. Although an individual living with AD/ADRD may experience mild symptoms at first, over time these symptoms will progress, ultimately requiring around‐the‐clock support.^1^ According to the Alzheimer's Association's 2025 Alzheimer's Disease Facts and Figures report, roughly 7.2 million Americans are living with Alzheimer's disease (AD), with that number expected to reach nearly 13.8 million by the year 2060.^1^ In an attempt to address this growing public health issue, federal funding for AD/ADRD research has seen a more than 7‐fold increase in the last decade. Today, federal funding for AD/ADRD research (National Institutes of Health [NIH]) exceeds $3.8 billion annually. This increased funding has led to more trials in the Alzheimer's space, with Cummings et al. reporting that there are 164 clinical trials testing 127 drugs for AD, requiring a total of 51,398 participants to populate all of the Phase 1, 2, and 3 trials as of January 2024.^2^ Further, the National Institute on Aging (NIA) is currently supporting almost 500 active AD/ADRD clinical trials, as well as 35 Alzheimer's Disease Research Centers (ADRCs) across the country.^3^, ^4^

There is a difference in the prevalence of AD between men and women, as well as according to racial and ethnic backgrounds and among sexual and gender minority (SGM) groups.^5^, ^6^, ^7^, ^8^, ^9^, ^10^, ^11^, ^12^, ^13^ Historical background, as well as cultural conditions, poses unique challenges in developing successful engagement, recruitment, and retention strategies. Successful engagement of all populations requires consideration of a person's economic stability, education, health care, built environment, and community, and how these factors change over a person's lifetime. Incorporating tools and resources that allow for ongoing connection with research participants and improving accessibility and awareness of research opportunities, including the number and variety of opportunities that exist, can contribute to more successful engagement, recruitment, and retention of research participants.^12^

The true number of AD/ADRD studies actively recruiting participants, including those that are observational in nature or that support the development of diagnostics, treatment, and improved care, far exceeds both the number of trials reported by Cummings et al. as well as the number currently being funded by the NIA. Now more than ever there is a critical need to encourage participation in AD/ADRD research. One such approach to addressing the need for research participation is the development of participant recruitment registries. These registries are tools that aid participants in identifying and screening for research opportunities, resulting in qualified referrals for researchers. A number of these registries exist in the AD/ADRD space, including some tailored to aid specific minoritized communities in identifying culturally and linguistically appropriate opportunities.^14^, ^15^, ^16^, ^17^, ^18^ Although differing in their approaches, registries require participants to complete various questionnaires or testing in order to match to opportunities. Although these registries can be highly effective at reducing screen fail, there are innate barriers within them that hinder participation. Langbaum et al. found that requiring specific tasks as part of the registry, such as providing biosamples or providing family contact information, can decrease intention to join a registry.^19^ By creating TrialMatch, the Alzheimer's Association sought to enhance awareness of AD/ADRD research using an accessible, reduced barrier approach.

## 2 METHODS

### 2.1 Platform overview

TrialMatch was launched in 2010 by the Alzheimer's Association. The TrialMatch platform used to conduct this analysis was CenterWatch iConnect, but beginning in January 2025, the TrialMatch platform transitioned to CareBox Connect. Anyone 18 or older is eligible to use TrialMatch. The service is available to anyone living with dementia, people with no current concerns about their memory, people who are caregivers for someone with dementia, as well as health care professionals or others searching on behalf of those living with dementia. TrialMatch is an awareness tool and does not directly enroll users into trials. To protect privacy, individual‐level data from questionnaire responses are not retained. Because TrialMatch is not a traditional registry, users do not provide consent when completing the TrialMatch questionnaire, but they are informed of the Privacy Policy. Individuals are given the opportunity to receive information about additional research opportunities by email as they become available. Users provide basic demographic information and an email address. Phone assistance through TrialMatch is available in both Spanish and English, as well as through a language line for those who need support in another language.

Studies are added to the database through two routes. Clinicaltrials.gov is routinely scrubbed for any actively recruiting trial that is related to dementia or brain health and monitored for any changes and updated accordingly. Researchers with studies not on clinicaltrials.gov are able to submit their opportunities to TrialMatch using an online submission portal. Submissions are evaluated for relevance and require ethics board approval before being added to the database. Opportunities added through researcher submission are reviewed quarterly for any updates or closures.

**RESEARCH IN CONTEXT**

1. **Systematic review**: The authors reviewed the literature using traditional (e.g., PubMed and Google Scholar) sources. Although recruitment science has made major strides in recent years, satisfying recruitment goals remains a major obstacle for dementia research. Relevant citations for the current state of dementia research are appropriately cited.
2. **Interpretation**: Our findings show that awareness platforms such as TrialMatch fill a gap in the recruitment pipeline for dementia research. Although registries can provide high‐quality referrals, awareness platforms have the potential for wider reach and present fewer barriers to users.
3. **Future directions**: This article highlights the success of platforms such as TrialMatch in raising awareness of dementia research studies. Future directions for this area of research include better measurement of the impact of this platform on the recruitment pipeline. The researchers hope to evaluate the conversion of platform use and study views into actionable and qualified study referrals.

### 2.2 TrialMatch awareness and engagement

The Alzheimer's Association has local offices in all 50 states and has thousands of staff and volunteers. Staff and volunteers are routinely trained on how to use the platform and promote the service using an extensive promotional toolkit, including print collateral tailored to different audiences, digital and print ads, talking points, and press releases. TrialMatch is linked on any Association web page containing information about research.

During the coronavirus disease 2019 (COVID‐19) pandemic, it became clear that users would need additional ways to engage with TrialMatch and research in the absence of in‐person opportunities. To address this, the team developed an enhanced ability to search for online or remote study opportunities. Users were able to toggle between studies that require in‐person visits or those that could be completed entirely online or over the phone. To enhance our ability to promote the TrialMatch service during this time, the team also implemented new collateral and promotional pieces that utilized contactless engagement methods such as QR codes and “text‐to‐join” functionality.

### 2.3 Product design

The TrialMatch tool was initially launched in 2010, and it required users to create an account, provide demographic and contact information, and complete individual questionnaires. Through community input, the team identified that this process presented multiple barriers to use for our constituents, such as hesitation around sharing personal information and the lengthy process to obtain match results. This feedback led to a full redesign of the user interface. In August 2020, the Alzheimer's Association launched a new version of the tool, which eliminated account creation and simplified the questionnaire experience. The redesign also added international studies.

### 2.4 User experience

Users can engage with TrialMatch through a self‐guided online portal, over the phone, and via email. TrialMatch has a team of navigators who are available Monday through Friday. These navigators are available by phone or email to answer questions and to assist users in finding qualified opportunities. Regardless of the method, users are presented with a brief series of questions that are designed to reduce the list of opportunities presented in their results. TrialMatch users are not required to create accounts or profiles within the system, provide any medical results or biosamples, participate in any testing, or provide contact information for themselves or a relative to receive results. Results are sorted automatically by proximity to the users, and users can further filter their results by study type, phase, and whether the study can be completed online or remotely. For matched opportunities, users are provided with contact information for each study site.

### 2.5 Data analysis

To protect user privacy, individual‐level data from questionnaire responses are not retained through TrialMatch. Engagement with TrialMatch was measured through web sessions, study referrals made through the website, calls, and voluntary email signups. Referral and voluntary email signup data were collected through CenterWatch iConnect, web traffic through Google Data Studio, and call volume through 8 × 8 Voice Over Internet Protocol.

TrialMatch is inclusive of both interventional and observational studies, providing a more comprehensive view of enrollment targets for all studies in the space. These data were reported by the study teams and gathered through clinicaltrials.gov or the online submission portal. Enrollment targets were evaluated overall, as well as after the exclusion of large national and international registries. Registries were excluded from additional analyses due to the significantly larger recruitment goals or lack of specific recruitment goals typically seen in such studies. Recruitment numbers were summarized according to study type and categorized based on recruitment goals or trial phase. Any data was due to missing, insufficient, or incomplete reporting by study teams.

## 3 RESULTS

### 3.1 Usage statistics

During the review period of August 2020 and June 2024, TrialMatch maintained a database of more than 700 studies, on average. TrialMatch received a total of 18,802 calls to its support line, 122,461 web sessions in which a user interacted with any component of the platform, and provided 17,725 referrals to studies in the database. A total of 4980 individuals consented to providing their contact information during this period, with the full user database consisting of 189,336 contacts. Emails sent to users averaged an open rate of 25%.

### 3.2 Enrollment targets

**FIGURE 1:**
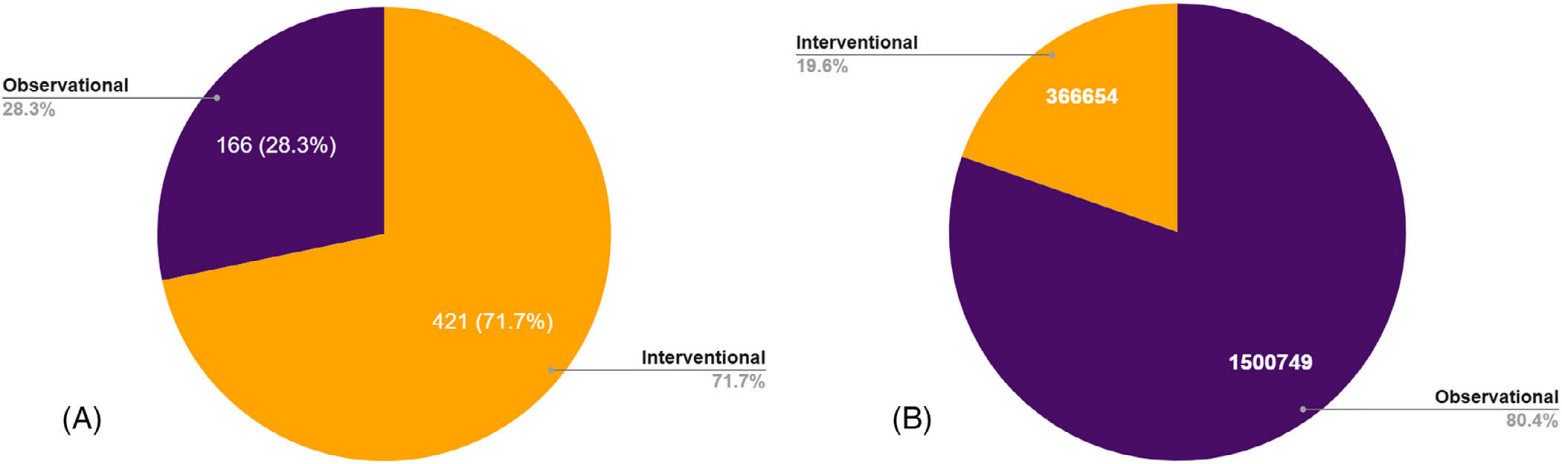
Distribution of observational and interventional studies in TrialMatch (A) and total enrollment required to complete all studies, by study type (B).

The database includes studies for AD, dementia, Lewy body dementia, mild cognitive impairment, vascular dementia, frontotemporal dementia, Parkinson's with dementia, and general brain health. Data on enrollment targets were available for 587 studies in the database. Of these, 166 (28.3%) of the studies are classified as observational and 421 (71.7%) as interventional (Figure 1). To fully populate all studies in the database, 1,867,403 participants are needed.

**FIGURE 2:**
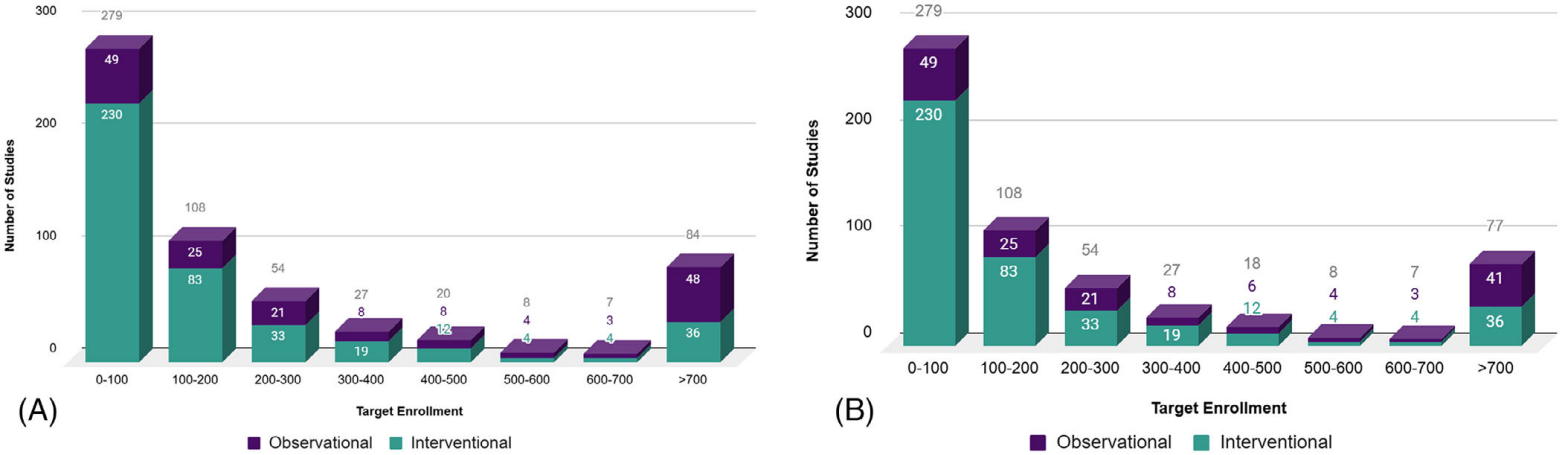
After the removal of large national and international registries, distribution of studies in TrialMatch by study type (A) and total enrollment required to complete all studies, by study type (B).

Despite comprising the majority of studies in the database, interventional studies required fewer participants (*n* = 366,654, 19.6%) compared to observational studies (*n* = 1,500,749, 80.4%) (Figure 1). Observational studies frequently targeted high enrollment estimates when compared to interventional studies (Figure 2).

**FIGURE 3:**
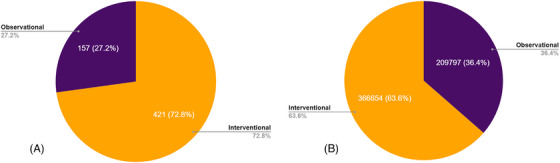
Distribution of interventional and observational studies by enrollment targets before (A) and after (B) removing large registries.

After removing large registries from the analysis, 578 studies remained in the database. Of these, 157 (27.2%) of the studies are classified as observational and 421 (72.8%) as interventional (Figure 3). In total, 576,451 volunteers are needed to fully meet the enrollment targets for these studies. Without the inclusion of these larger registries, interventional studies held the highest enrollment, with 366,654 (63.6%) volunteers needed compared to 209,793 (36.4%) for observational studies (Figure 3). Observational studies still frequently targeted high enrollment when compared to interventional studies (Figure 2).

### 3.3 Interventional trials by phase

**FIGURE 4:**
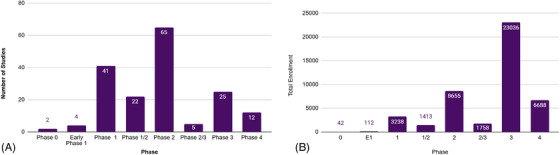
Distribution of interventional studies (A) and enrollment targets (B) in TrialMatch by study phase.

Information on the trial phase was available for 176 (41.8%) of the interventional studies. Of these, 45 (25.6%) were Phase 1; 22 (12.5%) Phase 1/2; 65 (36.9%) Phase 2; 5 (2.8%) Phase 2/3; 25 (14.2%) Phase 3; and 12 (6.8%) Phase 4 (Figure 4). Phase 2 and 3 studies comprised the largest proportion of the enrollment estimates, requiring 8655 and 23,036 participants, respectively (Figure 4).

## 4 DISCUSSION

The Alzheimer's Association's TrialMatch launched in 2010 as a user‐friendly tool for bringing together researchers, health care professionals, people living with dementia and their caregivers, and healthy volunteers in advancing dementia science. The objectives of this tool were to create a comprehensive, approachable, and central location for those impacted by dementia to identify and locate research opportunities, while simultaneously addressing one of the largest challenges facing dementia science: participant recruitment. As a result, TrialMatch engaged more than 400,000 unique constituents between 2010 and 2020 and assisted in raising awareness for hundreds of AD/ADRD and healthy aging studies. Since 2020, TrialMatch has averaged over 2600 web sessions and over 250 calls to our helpline monthly.

TrialMatch highlights the importance of engaging all communities in dementia research. Our data suggest that almost 1.9 million people are required to fulfill enrollment for ongoing studies for Alzheimer's and other dementias. Even after removing large international registries, the remaining research opportunities need more than 575,000 volunteers. High‐quality observational studies provide us with key insights into disease biology and progression, which form the foundation for future interventions. Of interest, most of the studies on TrialMatch have an enrollment goal of less than 100 participants. In addition, most of the interventional studies captured within TrialMatch are categorized as Phase 1 and 2 studies. This highlights the need to support these early‐stage research efforts to ensure that all potentially viable treatment options transition to the later stages of the clinical trials pipeline.

TrialMatch is an open website platform that does not require account creation or user verification. Although this makes the service more accessible and easy‐to‐use for individuals, it also makes it an easy target for spam and bots. In recent years, there has been a documented increase in bot attacks on health research, particularly research involving survey questionnaires. Although the reasons for these attacks are varied, they often are monetarily driven, with the bots pursuing the compensation provided by participating in research. This poses a unique challenge and increased burden for researchers trying to recruit eligible participants into their studies. Although it is not possible to completely eliminate this type of activity on TrialMatch, we sought to reduce this by requiring additional verification, as well as discouraging the inclusion of direct survey links within the content of a listing.

An additional limitation of TrialMatch is that users are not representative of the general population. Although users can choose to engage with TrialMatch over the phone, and a valid internet connection is not required to participate, an email address is needed to engage with a research study. This may be a barrier for individuals who do not have routine access to the internet, or for those who have concerns about sharing personal information over the phone or computer. Furthermore, although the Association aims to promote TrialMatch broadly, TrialMatch users may not be representative of the general population with regard to race, ethnicity, sex, gender, or age.

After over a decade of use, TrialMatch has demonstrated its capability as a research awareness tool. The data from TrialMatch provide the first ever estimates for clinical study enrollment for several biological causes of dementia. Due to the inclusive nature of the service, TrialMatch is able to highlight the critical need for inclusion in clinical trials for all causes of dementia. Unlike similar tools, such as traditional registries, TrialMatch requires fewer barriers to participation. Although reducing barriers to use enables the tool to reach a broader audience, it hinders our ability to track users through the recruitment pipeline. In its current state, TrialMatch is unable to track whether a referral results in a study enrollment. User engagement continues to remain a priority for TrialMatch, and future changes to the service aim to more effectively engage and reach new audiences. Additional focus areas for the future include enhanced tracking from first interaction with TrialMatch to study enrollment. Starting in 2025, TrialMatch began providing optional follow‐up to users and will capture data on whether users were able to connect with research teams, whether they were able to enroll in research opportunities, and what barriers they encountered throughout the process. In gathering these data, as well as additional demographic and use data, the Alzheimer's Association should be able to improve the effectiveness of the platform and activate strategies for awareness, particularly in communities not yet served by the tool.

## CONFLICT OF INTEREST STATEMENT

The authors declare no conflicts of interest. Any author disclosures are available in the supporting information.

## FUNDING INFORMATION

There is no funding to disclose for this manuscript.

## CONSENT STATEMENT

Informed consent was not necessary for this article, as individual‐level data were not obtained or analyzed. All data presented in this manuscript are aggregate data collected from a publicly available clinical trial matching tool.

## Supporting information



Supporting Information

## References

[1] Alzheimer's Association . 2025 Alzheimer's disease facts and figures. Alzheimers Dement. 2025;21(4):e70235. 10.1016/j.jalz.2016.03.001 27570871

[2] Cummings J , Zhou Y , Lee G , Zhong K , Fonseca J , Cheng F . Alzheimer's disease drug development pipeline: 2024. Alzheimers Dement. 2024;10(2):e12465. 10.1002/trc2.12465 PMC11040692 38659717

[3] NIA‐funded active Alzheimer's and related dementias clinical trials and studies | National Institute on Aging. (n.d.). https://www.nia.nih.gov/research/ongoing‐AD‐trials

[4] Find an alzheimer's disease research center | National Institute on Aging (n.d.). https://www.nia.nih.gov/health/clinical‐trials‐and‐studies/alzheimers‐disease‐research‐centers

[5] Rajan KB , Weuve J , Barnes LL , McAninch EA , Wilson RS , Evans DA . Population estimate of people with clinical AD and mild cognitive impairment in the United States (2020‐2060). Alzheimers Dement. 2021;17(12):1966–1975. 34043283 10.1002/alz.12362 PMC9013315

[6] Rajan KB , Weuve J , Barnes LL , Wilson RS , Evans DA . Prevalence and incidence of clinically diagnosed Alzheimer's disease dementia from 1994 to 2012 in a population study. Alzheimers Dement. 2019;15(1):1–7. 30195482 10.1016/j.jalz.2018.07.216 PMC6531287

[7] Manly JJ , Jones RN , Langa KM , et al. Estimating the prevalence of dementia and mild cognitive impairment in the US: the 2016 Health and Retirement Study Harmonized Cognitive Assessment Protocol Project. JAMA Neurol. 2022;79(12):1242–1249. 36279130 10.1001/jamaneurol.2022.3543 PMC9593315

[8] Potter GG , Plassman BL , Burke JR , et al. Cognitive performance and informant reports in the diagnosis of cognitive impairment and dementia in African Americans and whites. Alzheimers Dement. 2009;5(6):445–453. 19896583 10.1016/j.jalz.2009.04.1234 PMC2805266

[9] Gurland BJ , Wilder DE , Lantigua R , et al. Rates of dementia in three ethnoracial groups. Int J Geriatr Psychiatry. 1999;14(6):481–493. 10398359

[10] Mielke MM , Aggarwal NT , Vila‐Castelar C , et al. Consideration of sex and gender in Alzheimer's disease and related disorders from a global perspective. Alzheimers Dement. 2022;18(12):2707–2724. doi:10.1002/alz.12662 35394117 10.1002/alz.12662 PMC9547039

[11] Liu H , Hsieh N , Zhang Z , Zhang Y , Langa KM . Same‐sex couples and cognitive impairment: evidence from the health and retirement study. J Gerontol B. 2021;76(7):1388–1399. 10.1093/geronb/gbaa202 PMC8499509 33211882

[12] Hsieh N , Liu H , Lai WH . Elevated risk of cognitive impairment among older sexual minorities: do health conditions, health behaviors, and social connections matter?. Gerontologist. 2021;61(3):352–362. 32951038 10.1093/geront/gnaa136 PMC8023357

[13] Dragon CN , Guerino P , Ewald E , Laffan AM . Transgender Medicare beneficiaries and chronic conditions: exploring fee‐for‐service claims data. LGBT health. 2017;4(6):404–411. 29125908 10.1089/lgbt.2016.0208 PMC5731542

[14] Ta Park VM , Meyer OL , Tsoh JY , et al. The Collaborative Approach for Asian Americans and Pacific Islanders Research and Education (CARE): a recruitment registry for Alzheimer's disease and related dementias, aging, and caregiver‐related research. Alzheimers Dement. 2023;19(2):433–443. 35420258 10.1002/alz.12667 PMC9562598

[15] High NM , Kettenhoven CL , Graf H , Reiman EM , Tariot PN , Langbaum JB , (2020). The Alzheimer's Prevention Registry: accelerating recruitment and enrollment into Alzheimer's‐focused studies. In 2020 Alzheimer's Association International Conference . ALZ. 10.14283/jpad.2020.31 PMC7534299 32920626

[16] Weiner MW , Nosheny R , Camacho M , et al. The Brain Health Registry: an internet‐based platform for recruitment, assessment, and longitudinal monitoring of participants for neuroscience studies. Alzheimers Dement. 2018;14(8):1063–1076. 29754989 10.1016/j.jalz.2018.02.021 PMC6126911

[17] Chadiha LA , Washington OG , Lichtenberg PA , Green CR , Daniels KL , Jackson JS . Building a registry of research volunteers among older urban African Americans: recruitment processes and outcomes from a community‐based partnership. Gerontologist. 2011;51(suppl_1):S106–S115. 21565812 10.1093/geront/gnr034 PMC3092980

[18] Langbaum JB , High N , Nichols J , Kettenhoven C , Reiman EM , Tariot PN . The Alzheimer's Prevention Registry: a large internet‐based participant recruitment registry to accelerate referrals to Alzheimer's‐focused studies. J Prev Alzheimers Dis. 2020;7(4):242–250. 32920626 10.14283/jpad.2020.31 PMC7534299

[19] Langbaum JB , Maloney E , Hennessy M , et al. How intention to join an Alzheimer's participant recruitment registry differs by race, ethnicity, sex, and family history: results from a national survey of US adults. Alzheimers Dement. 2023;19(12):5399–5406. 37204220 10.1002/alz.13126 PMC10657330

